# Plasma levels of IL-6 and IL-10 in septic patients at admission and during follow-up and association with clinical outcomes

**DOI:** 10.1186/cc10167

**Published:** 2011-06-22

**Authors:** RT Costa, MKC Brunialti, F Machado, E Silva, O Rigato, R Salomão

**Affiliations:** 1 Division of Infectious Diseases, Escola Paulista de Medicina, Federal University of São Paulo, São Paulo - SP, Brazil; 2 Hospital Sírio-Libanês, São Paulo - SP, Brazil; 3 Discipline of Anesthesiology, Escola Paulista de Medicina, Federal University of São Paulo, São Paulo - SP, Brazil; 4 Israelita Albert Einstein Hospital, São Paulo - SP, Brazil

## Introduction

Sepsis is a systemic inflammatory syndrome triggered by infection. It has been recognized that a dynamic interaction between proinflammatory and anti-inflammatory response is present in this syndrome, which is balanced by as yet unknown mechanisms. We and others showed that inflammatory cytokines are upregulated in the early phase and downregulated in the late phases of sepsis, while anti-inflammatory cytokines are preserved. However, there are few data about the dynamics of these cytokines during follow-up of patients and their relation with clinical outcome. The aim of this study was to evaluate the plasma levels of a proinflammatory, IL-6, and an anti-inflammatory, IL-10, cytokine in septic patients.

## Methods

This prospective study included 53 septic patients (SP) and 29 healthy volunteers (HV) as a control group. Patients were admitted to the intensive care units of São Paulo, Sirio-Libanes and Israelita Albert Einstein hospitals. Samples were collected during the first 48 hours of organ dysfunction or sepsis (D0). A second sample was collected after 7 days from 35 SP (D7). The plasma levels of cytokines were measured using the cytometric bead array method (limit detection 2.0 pg/ml) by flow cytometry.

## Results

IL-6 and IL-10 plasma levels were higher in SP (median 170.8 pg/ml, range 3.53 to 16,028.52 pg/ml; and median 6.6 pg/ml, range 0.0 to 1,698.92 pg/ml, respectively) than HV (median 2.3 pg/ml, range 0.0 to 19.92 pg/ml for IL-6; and median 2.4 pg/ml, range 0.0 to 12.7 pg/ml for IL-10) (*P *= 0.0001 and *P *= 0.007, respectively). Plasma levels of IL-6 and IL-10 at D7 were not significant different from those at D0 (*P *= 0.85 and *P *= 0.59, respectively). IL-6 and IL-10 admission plasma levels were higher in nonsurvivors (median 284.76 pg/ml, range 9.16 to 16,028.52 pg/ml; and median 17.6 pg/ml, range 0.0 to 1,698.92 pg/ml, respectively) than in survivors (median 103.57, range 3.53 to 9,745.43 pg/ml; and median 9.91 pg/ml, range 0.0 to 313 pg/ml; *P *= 0.02 and *P *= 0.003, respectively).

## Conclusion

Our results show that both proinflammatory and anti-inflammatory cytokines are detected during sepsis and a higher level of both cytokines at admission is associated with worst outcomes.

